# Socioeconomic Disparities in Brachial Plexus Surgery: A National Database Analysis

**DOI:** 10.1097/GOX.0000000000002118

**Published:** 2019-02-05

**Authors:** Alexandra Bucknor, Anne Huang, Winona Wu, Aaron Fleishman, Sabine Egeler, Anmol Chattha, Samuel J. Lin, Matthew L. Iorio

**Affiliations:** From the * Department of Surgery, Division of Plastic and Reconstructive Surgery, Beth Israel Deaconess Medical Center, Harvard Medical School, Boston, Mass.; † Department of Surgery, Beth Israel Deaconess Medical Center, Harvard Medical School, Boston, Mass.; ‡ Department of Surgery, Division of Plastic and Reconstructive Surgery, University of Colorado, Anschutz Medical Center, Aurora, Colo.

## Abstract

**Background:**

Brachial plexus injuries have devastating effects on upper extremity function, with significant pain, psychosocial stress, and reduced quality of life. The aim of this study is to identify socioeconomic disparities in the receipt of brachial plexus repair in the emergent versus elective setting, and in the use of supported services on discharge.

**Methods:**

Analysis of the Healthcare Cost and Utilization Project National Inpatient Sample Database was performed for the years 2009–2014. Adults with brachial plexus injury with or without nerve repair were identified; patient and hospital specific factors were analyzed.

**Results:**

Overall, 6,618 cases of emergent brachial plexus injury were retrieved. Six hundred sixty cases of brachial plexus repair were identified in the emergency and elective settings over the study period. Of the 6,618 injured, 153 (2.3%) underwent nerve surgery during the admission. Patients undergoing repair in the elective setting were more likely to be white males with private insurance. Patients treated in the emergency setting were more likely to be African American and in the lowest income quartile. Significant differences were also seen in supported discharge: more likely males (*P* < 0.001), >55 years of age (*P* < 0.001), white (*P* < 0.001), with government-based insurance (*P* < 0.001).

**Conclusions:**

There are significant disparities in the timing of brachial plexus surgery. These relate to timing rather than receipt of nerve repair; socioeconomically advantaged individuals with private insurance in the higher income quartiles are more likely to undergo surgery in the elective setting and have a supported discharge.

## INTRODUCTION

Brachial plexus injuries can have devastating effects on upper extremity function, with significant pain, psychosocial stress, and reduced quality of life.^1,2^ Although there is still debate over the optimal timing of surgical intervention, earlier intervention has been associated with improved outcomes in certain circumstances.^3,4^

However, the timing of these interventions can be frequently delayed by factors other than the clinical setting, such as accessibility to care, or the availability of regional resources to perform the specialist surgery and rehabilitate appropriately afterwards.^5^ Previous research has identified that lack of finances and access to transportation are limiting agents in patients accessing care in the United States.^6^ Patients may find themselves requiring functional support after surgery, and may require a supported discharge to a nursing facility; these needs may also influence access to and use of services, and it may be that the same socioeconomic factors may also hamper access to supported discharge facilities.

Understanding factors that influence the timing of brachial plexus surgery after injury and outcomes may help to identify risk factors for suboptimal management and complications, and the potential need for implementing a structured surgical approach to provide optimal patient care.^7^ Using the Healthcare Cost and Utilization Project Kids’ Inpatient Database, Squitieri et al.^7^ found that patients with private insurance were significantly more likely to undergo nerve reconstruction in cases of neonatal brachial plexus injury. However, the variability of socioeconomic factors in the adult, traumatic brachial plexus injury population has not been evaluated for similar discrepancies.

The aim of this article is 3-fold: (1) to analyze national data to ascertain whether there are disparities in the receipt of adult brachial plexus repair in the emergent verses elective setting; (2) to determine whether there are disparities in the receipt of supported discharge after brachial plexus injury in the acute setting; and (3) to evaluate whether brachial plexus repair in the emergency setting influences the need for supported discharge.

## METHODS

### Database and Cohort Selection

A retrospective analysis of the Healthcare Cost and Utilization Project National Inpatient Sample (NIS) Database from the Agency for Healthcare Research was performed for the years 2009–2014. The NIS includes data from >8 million discharge abstracts annually, approximating a 20% sample of all discharges, including nonfederal, short-term, general, and other specialty hospitals, including public hospitals and academic institutions.^8^ The NIS is the largest, publicly available all-payer inpatient database in the United States.

Data were extracted using International Classification of Diseases, 9th Revision, Clinical Modification (ICD-9-CM) codes. Adults 18 years of age and over with diagnosis of brachial plexus injury were retrieved (ICD-9-CM diagnosis code: 9543—brachial plexus injury). Procedural data for nerve repair were retrieved using ICD-9-CM codes that have been used in previous research.^7^

### Outcome Variables

Data were collected on patient and hospital characteristics, including gender, age in years (18–34, 35–54, 55+), race (white, African American, Hispanic, other, missing), comorbidities occurring in ≥5% of cases, hospital bed size (small, medium, and large), hospital teaching status (collapsed into rural/urban nonteaching and urban teaching for analysis), hospital region (Northeast, Midwest, South, West), median household income quartile (1 being the lowest), and primary payer (government/state-based, private, other). We also collected data on concomitant injury patterns (orthopedic, thoracoabdominal, or head injury), and discharge status using the “DISPUNIFORM” variable (determining whether or not the patient required supported care on discharge; this was defined as any discharge destination that was not home or self-care).

### Statistical Analysis

Data were analyzed using IBM SPSS Statistics for Macintosh, Version 24 (IBM Corp, Armonk, NY), and statistical significance was taken when *P* < 0.05. Patient and hospital-level characteristics were compared in those undergoing nerve repair in the emergency or elective setting, using Pearson chi-square or Fisher exact tests, as appropriate. Patient and hospital characteristics and discharge status were also compared between those receiving nerve surgery in the emergency setting and those not receiving surgery. Variables subsequently underwent binary logistic regression modeling to evaluate factors affecting the likelihood of supported care on discharge, compared with discharge home. Factors that were significant on univariable analysis or deemed clinically relevant were included in the multivariable model. Results are presented as odds ratios (ORs) with 95% CIs.

## RESULTS

### Brachial Plexus Repair: Elective Versus Emergency Patients

Over the 6-year period, 660 cases of brachial plexus repair were captured in both the emergency and elective settings. We reviewed the patient and hospital-level characteristics of those undergoing surgery in the emergency setting compared with the elective setting (Table 1). There were significant differences in gender (*P* < 0.01), age (*P* < 0.001), ethnicity (*P* < 0.001), insurance (*P* < 0.001), and household income (*P* < 0.001). Patients undergoing repair in the elective setting were relatively more likely to be white (64.0% versus 45.1%) males (90.3% versus 80.4%) with private insurance (55.8% versus 32.0%). Patients treated in the emergency setting were more likely to be African American (19.6 versus 9.7%) and in the lowest income quartile (39.2% versus 21.9%).

**Table 1.**
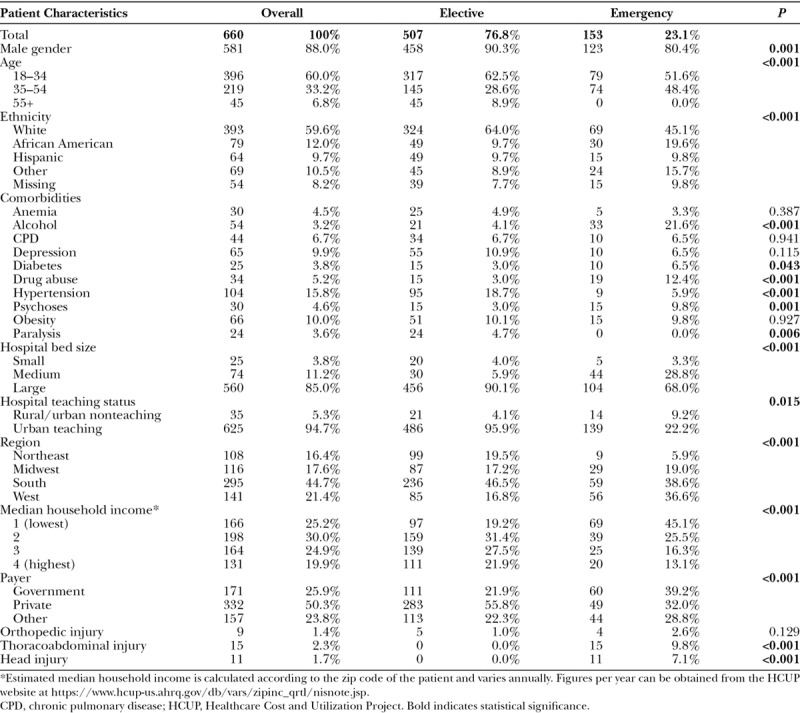
Comparison of Characteristics of Those Undergoing Brachial Plexus Repair in the Emergency or Elective Setting

### Emergency Admission With Brachial Plexus Injury

Having identified these demographic differences in characteristics between patients undergoing emergent and elective repairs, we then focused our analysis on characteristics of those who received a supported discharge after diagnosis of brachial plexus injury in the emergency setting, compared with those who did not. Overall, 6,618 cases of emergent brachial plexus injury were retrieved. Of these, 153 (2.3%) underwent nerve repair surgery during the admission.

There were significant differences in characteristics between those receiving supported discharge and those not, as detailed in Table 2. Those receiving supported discharge were more likely to be male (65.6% versus 34.4%; *P* < 0.001), relatively more likely to be >55 years of age (41.8% versus 19.0%; *P* < 0.001), white (64.7% versus 57.0%; *P* < 0.001), with government-based insurance (45.6% versus 29.4%; *P* < 0.001). Patients receiving supported discharge were more likely to have anemia (*P* < 0.001), chronic pulmonary disease (*P* = 0.023), depression (*P* = 0.003), diabetes (*P* < 0.001), hypertension (*P* < 0.001), hypothyroidism (*P* < 0.001), obesity (*P* < 0.001), and concomitant psychoses (*P* < 0.01). Patients receiving supported discharge were also more likely to be paralyzed (9.7% versus 4.5%; *P* < 0.001). There were also significant differences in hospital bed size (*P* < 0.001) and region (*P* < 0.001).

**Table 2.**
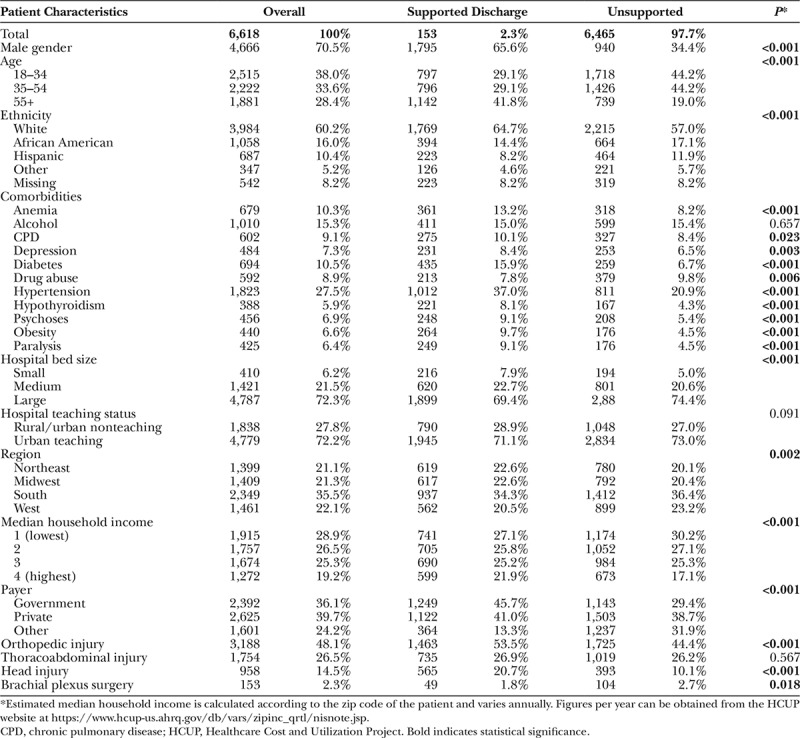
Comparison of Outcomes in Those Admitted With an Emergency Brachial Plexus Injury Between Those Requiring Supported Discharge or Not

On unadjusted analysis, there were lower rates of brachial plexus surgery in those requiring supportive care on discharge compared with those who were discharged home (1.8% versus 2.7%; *P* = 0.018). On multivariable analysis, there were many factors that affected the likelihood of supported discharge, as shown in Table 3. Notably, brachial plexus surgery in the emergency setting was associated with an increased need for supported discharge, after adjusting for other patient and hospital-level characteristics (OR, 1.804; CI, 1.225–2.657). Highest quartile income also increased the likelihood of supported discharge (OR, 1.354; CI, 1.137–1.613).

**Table 3.**
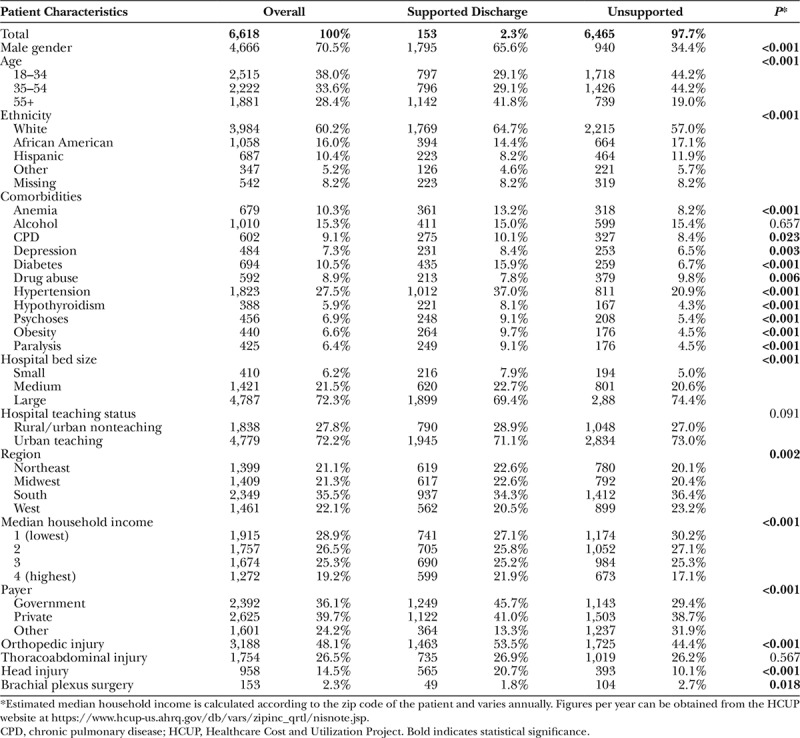
Results of Multivariable Binary Logistic Regression: Factors Associated With Supported Discharge in the Emergent Brachial Plexus Injury

## DISCUSSION

This study examined the NIS from 2009 to 2014 to evaluate potential differences in the characteristics of those who undergo brachial plexus surgery during an emergency or elective admission and factors affecting likelihood of requiring supported discharge in those with an acute brachial plexus injury. Overall, our results suggest that there are socioeconomic factors affecting the timing of brachial plexus surgery. Moreover, patients undergoing brachial plexus surgery in the acute setting are less likely to be discharged home in a self-caring status, but those who do received supported care on discharge are more likely to be in the highest income quartile.

There are clear indications for delayed repair, including gravity of associated injuries and less-severe brachial plexus injuries with a favorable prognosis.^9^ The exact timing of surgery after other brachial plexus injuries is controversial. Proponents of immediate reconstruction believe that axonal transection leads to neuronal degeneration due to loss of neurotrophins, and that delayed repair is associated with irreversible neuronal death.^9^ Delayed surgery is also more technically challenging because of scarred tissue planes and recoil of ruptured nerves.^9^ In contrast, proponents of delayed reconstruction believe that waiting 2–4 months can be essential for accurate assessment of the injury using magnetic resonance imaging and electrodiagnostic studies and for optimizing surgical planning.^10^

However, our results suggest that it is more than clinical reasoning alone influencing the timing of care: white males with private insurance were far more likely to be treated in the elective setting than African American patients or those in the lowest income quartile. Of course, it may be that injury patterns are different in these distinct demographic groups. Analysis of the U.S. National Trauma Data Bank has shown that African American and Hispanic patients, and those who are uninsured, are not only more likely to be the subject of high-energy penetrating trauma than white or insured patients, but also more likely to die as a result of their injuries.^11^ The authors also found that after controlling for anatomical and physiological injury severity, in addition to mechanism of injury, the disparities persisted—suggesting that injury pattern alone does not fully explain the findings. Therefore, we must also consider that other factors, such as patient education and resources, play a role.

According to work published by Thomas and Penchansky,^12^ there are 5 distinct components to consider when examining access to health care: affordability, accommodation, availability, accessibility, and acceptability.^13^ A study of 12 patients with brachial plexus injury found that the biggest barriers to surgery were lack of insurance coverage, insufficient information regarding treatment options, and delayed diagnosis.^14^ The reverse, therefore, may also be true: it may be that the more socioeconomically advantaged individuals identified in the present study have a greater awareness of the need for specialist input, coupled with the financial resources to seek out care at a specialist nerve center, thus delaying their care until they can find a surgeon of their choosing. Clinicians and hospital systems should consider the socioeconomic barriers to ethnic minority, low-income patients with brachial plexus injury that affect their access to specialist services. Shafi et al.^15^ published results of U.S. population study examining factors affecting placement into rehabilitation after traumatic brain injury.^11^ Results showed that, even after adjusting for injury severity and insurance status, ethnic minority patients were less likely to be placed into rehabilitation on discharge than non-Hispanic white patients. The authors postulated several reasons for this, including the prohibitive effects of cost, inadequate identification of rehabilitation requirements, geographic distance prohibiting travel for patients and families, or lack of certain services, such as translators.^11^

We also observed disparities in the receipt of supported discharge for those with a diagnosis of brachial plexus injury in the acute setting: those in the highest income quartile were more likely to received supported care on discharge. Interestingly, those who underwent brachial plexus repair in the emergent setting were also more likely to require supported discharge. The increase in patients being discharged to a supported facility rather than home as found in the present study is perhaps surprising because it is widely accepted that traumatic brachial plexus injury benefits from early surgical exploration and repair.^9^ Although it is difficult based on the heterogeneity of the research population to determine individual injury patterns and associated injuries, indications for an early repair commonly include concomitant arterial injury or penetrating trauma.^10^ Whereas, closed or blunt injures may have a higher rate of deferred repair in the elective setting. As discussed earlier, it is highly likely that injury severity, which is not captured by the NIS, plays an important role in our observations: those undergoing brachial plexus repair in the acute setting are perhaps more likely to have had injuries where there was no doubt about the diagnosis as a result of increased severity of the injury. Moreover, it is important to highlight that the severity of the underlying injury might lead to increased need for supported discharge, which we could not include in our analysis. We attempted to adjust for this by factoring concomitant major injury, but future studies may benefit from an assessment of brachial plexus injury severity.

Bringing these findings together, our results suggest that race, insurance status, and income may affect timing of brachial plexus surgery in the adult population, and that income may affect receipt of supported discharge.

Moving forward, when considering brachial plexus service provision, clinicians and hospital systems should identify what the specific barriers to these groups are and find ways of circumnavigating them. For example, the inclusion of language and culturally appropriate educational material has been found to be of key importance in ensuring equitable access to healthcare services for minority ethnic populations.^11^ Readily available translation services should be provided. Consideration of ways to address costs that may prohibit access to rehabilitative services may be beneficial, such as sponsored hospital transport, or telemedicine clinics enabling specialist input in remote areas.

There are a variety of charities and patient support groups for people with brachial plexus injuries in the United States.^16^ There are those that focus on connecting patients with other individuals who have had a similar experience to create a support network for those affected. Others serve to educate on the injuries and management strategies in the short and long term. In addition to these, some provide information on adaptive equipment that may help the individual perform their activities of daily living more independently. Information on legal proceedings that may be relevant is also highlighted, including relevant disability acts and provisions that may be required in certain situations. Health care providers may consider ways to increase the collaboration with these charitable organizations to improve the overall care provided to this patient group.

### Limitations

There are several limitations of this study. The retrospective analysis of administrative data may be prone to human error, as the reliability and validity of the data depend on ICD-9-CM coding. There is no specific code for brachial plexus nerve repair and so we rely on surrogate codes that have been used by previous authors, but these may encompass other concomitant nerve surgeries during the same admission. As we have highlighted, one of the most important limitations is that we did not have data on injury severity or mechanism of injury. The lack of information specifically relating to clinical presentation, referral patterns, and the rationale behind the treatment decision-making limits our understanding of the whole picture. Finally, this dataset does not provide information on outpatient visits or referrals, making it difficult to assess long-term patient outcomes.

## CONCLUSIONS

There are socioeconomic disparities in the timing of brachial plexus surgery: socioeconomically advantaged individuals with private insurance in the higher income quartiles are more likely to undergo surgery in the elective setting. Moreover, patients undergoing brachial plexus surgery in the acute setting are more likely to require supported discharge. Further research should seek to fully elucidate any disparities that may exist, so that equitable provision of brachial plexus services may be possible.
